# CO Responses of Sensors Based on Cerium Oxide Thick Films Prepared from Clustered Spherical Nanoparticles

**DOI:** 10.3390/s130303252

**Published:** 2013-03-08

**Authors:** Noriya Izu, Ichiro Matsubara, Toshio Itoh, Takafumi Akamatsu, Woosuck Shin

**Affiliations:** National Institute of Advanced Industrial Science and Technology (AIST), Advanced Manufacturing Research Institute, 2266-98 Anagahora, Shimo-Shidami, Moriyama-ku, Nagoya 463-8560, Japan; E-Mails: matsubara-i@aist.go.jp (I.M.); itoh-toshio@aist.go.jp (T.I.); t-akamatsu@aist.go.jp (T.A.); w.shin@aist.go.jp (W.S.)

**Keywords:** carbon monoxide, cerium oxide, gas sensor, core-shell nanoparticles, resistive-type sensor

## Abstract

Various types of CO sensors based on cerium oxide (ceria) have been reported recently. It has also been reported that the response speed of CO sensors fabricated from porous ceria thick films comprising nanoparticles is extremely high. However, the response value of such sensors is not suitably high. In this study, we investigated methods of improving the response values of CO sensors based on ceria and prepared gas sensors from core-shell ceria polymer hybrid nanoparticles. These hybrid nanoparticles have been reported to have a unique structure: The core consists of a cluster of ceria crystallites several nanometers in size. We compared the characteristics of the sensors based on thick films prepared from core-shell nanoparticles with those of sensors based on thick films prepared from conventionally used precipitated nanoparticles. The sensors prepared from the core-shell nanoparticles exhibited a resistance that was ten times greater than that of the sensors prepared from the precipitated nanoparticles. The response values of the gas sensors based on the core-shell nanoparticles also was higher than that of the sensors based on the precipitated nanoparticles. Finally, improvements in sensor response were also noticed after the addition of Au nanoparticles to the thick films used to fabricate the two types of sensors.

## 1. Introduction

Exposure to CO in a closed environment for 1 to 3 min, even at concentrations as low as 1%–2%, can prove fatal. Members of emergency response teams usually do not carry the equipment necessary for detecting CO levels when they respond to an emergency such as a rescue. If the CO concentration in an enclosed area is high, it can pose a danger to anyone responding to such emergencies if they fail to detect the level of CO present. In such situations, a portable CO sensor with an alarm can be invaluable in alerting one to the danger posed by the high CO level and allow one to take necessary protective measures. To ensure this, the CO sensor should have a high response speed as that would help limit exposure to CO. Additionally, since CO sensors are also used to monitor the combustion of natural or petroleum gas, sensors with high response speeds are essential for the precise control of these combustion processes. Hence, there is a pressing demand for fast CO sensors.

A number of different types of CO sensors have been designed [1–5]. Recently, CO sensors based on cerium oxide have also been reported [6,7]. The response speeds of these CO sensors, which comprised porous thick films made of cerium oxide nanoparticles, were extremely high. For example, the response time for detecting 5,000 ppm of CO was only a few seconds. The thick films used in such CO sensors had been prepared from ceria nanoparticles fabricated via precipitation [6,7]. In addition, it has been reported that CO sensors formed using thick films fired at 950 °C also exhibited higher response speeds [7]. However, there is still room for further improvements in the response values of such CO sensors.

A general approach for improving the response value and sensitivity of gas sensors has been to fabricate sensors from materials that comprise macrosized pores and clusters [8–10]. However, cerium oxide based gas sensors with such structures have not yet been fabricated as it is very difficult to form thick layers of cerium oxide consisting of such structures from conventionally fabricated nanoparticles.

There have been recent reports on the fabrication of core-shell cerium oxide polymer hybrid nanoparticles [11–14]. Core-shell cerium oxide polymer hybrid nanoparticles have a unique structure: the cores of these hybrids consist of clusters of cerium oxide crystallites several nanometers in size, with the core size distribution being very narrow. Such clusters 100 nm in size or larger can result in the formation of macrosized pores. Gas sensors that use these clusters of nanosized crystallites as the sensing material are expected to exhibit increased response values.

In this study, we prepared CO sensors using core-shell cerium oxide polymer hybrid nanoparticles as the sensing material and characterized the sensors, while also comparing their performance with that of CO sensors based on conventionally fabricated nanoparticles (*i.e.*, those produced via precipitation).

## 2. Experimental

The cerium oxide nanoparticles were prepared via a polyol-based method. Polyvinyl pyrrolidone (PVP, 120 kg·m^−3^; Sigma-Aldrich, St. Louis, MO, USA) and Ce(NO_3_)_3_·6H_2_O (0.6 kmol·m^−3^; Kojundo Chemical Laboratory, Sakado, Japan) were added to ethylene glycol (Wako Pure Chemical Industries, Osaka, Japan) and stirred. The PVP used had an average molecular weight of 10,000 (manufacturer's specification). The mixture was reflux heated at a set temperature (190 °C) for 120 min, with the set temperature being the temperature of the heater and not of the mixture. On the completion of the refluxing process, a dispersion sol was obtained. Any unreacted material and excess PVP were removed from the as-prepared sol by centrifugation. Next, the resultant residue was washed with water and ethanol and allowed to dry at room temperature, yielding a powder consisting of “core-shell nanoparticles” of cerium oxide.

Next, for comparison, nanoparticles of cerium oxide were also fabricated via a precipitation-based method. First, Ce(NO_3_)_3_·6H_2_O was added to water. Next, aqueous ammonia (25%) was added to the solution, resulting in the formation of a precipitate. This precipitate was filtered and a white gel was obtained. This white gel was then mixed with a commercial carbon black powder. The mixture was dried at 70 °C and calcined at 900 °C, resulting in a powder of “precipitated nanoparticles.” The method for preparing the precipitated nanoparticles has been described in greater detail elsewhere [15,16]. The two powders were mixed with an organic binder consisting of terpineol and ethyl cellulose to form pastes.

Pt electrodes and a Pt heater were deposited on the front and back sides of alumina substrates, respectively, via screen-printing using a Pt paste, and the substrates were subsequently fired. The two pastes, containing the core-shell nanoparticles and the precipitated nanoparticles, respectively, were then used to coat the Pt-electrode sides of the substrates via screen printing.

The substrates with the screen-printed thick films of the two pastes were calcined at 500 °C for 5 h in air and fired at 900–1,000 °C for 2 h in air. This yielded resistive-type gas sensors based on thick films of cerium oxide.

In a few cases, an Au colloid was added to the thick films in order to increase the response values of the corresponding sensors [17]. This was done by placing a few drops of an Au colloid dispersion onto the surfaces of the cerium oxide thick films using a syringe, drying the drops at 60 °C, and then heating the films at 400 °C for 2 h in air. The Au concentration in these thick films was approximately 4 wt.%.

Next, the fabricated sensors were characterized. This was done in a test chamber. The sensors were heated using the heaters on their back sides. Both compressed air containing CO and CO-free air were alternatively introduced into the chamber. The resistance between the Pt electrodes of the sensors was measured as a function of time using a digital multimeter; the details of these measurements have been reported previously [7]. In this study, the response value, *S*, of a sensor was defined as:

(1) $$S=R_{\text{air}}/R_{CO}$$

where *R*_CO_ and *R*_air_ are the resistances between the Pt electrodes of the sensors in air containing CO and in CO-free air, respectively.

The 90% response time (*t*_90,res_) and 90% recovery time (*t*_90,rec_) were defined as the time required for (*R*_air_–*R*)/(*R*_air_–*R*_CO_) to reach a value of 0.90 when the gas tested was changed from CO-free air to air containing CO and for (*R*-*R*_CO_)/(*R*_air_-*R*_CO_) to reach a value of 0.90 when the tested gas was changed from air containing CO to CO-free air, respectively, with *R* being the instantaneous value of the sensor resistance. The specific surface areas of the thick films were determined via the Brunauer-Emmett-Teller (BET) method (NOVA4200e, Quantachrome Instruments, Boynton Beach, FL, USA).

## 3. Results and Discussion

**Figure 1.**
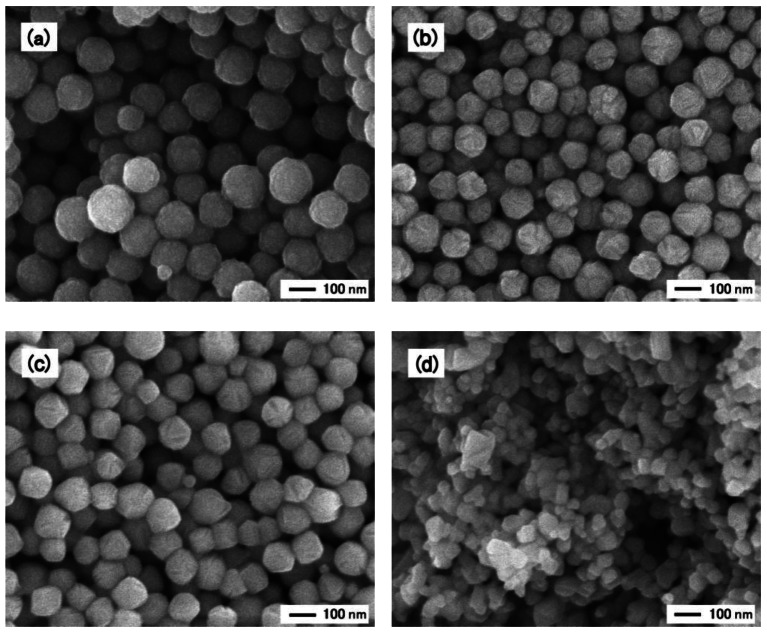
SEM images of the cerium oxide nanoparticles and thick films used in this study. (**a**) the as-prepared cerium oxide nanoparticles, (**b**) a thick film prepared from the core-shell nanoparticles, after it had been fired at 900 °C, (**c**) a thick film prepared from the core-shell nanoparticles, after it had been fired at 1,000 °C, and (**d**) a thick film prepared from the precipitated nanoparticles, after it had been fired at 950 °C.

Figure 1 shows SEM images of the core-shell nanoparticles prepared by the polyol-based method and the corresponding thick films after they had been fired at 900–1,000 °C. The average size of the nanoparticles used in this study was determined from Figure 1(a) and was found to be approximately 145 nm.

Using X-ray diffraction (XRD) analyses, it was confirmed that the nanoparticles were indeed of cerium oxide and that the crystallites of the nanoparticles ranged in size from 2 to 8 nm; this was in keeping with the results of previous studies [11,14]. In addition, the results of thermogravimetric analysis (TGA) and Fourier transform infrared (FT-IR) spectroscopy were also similar to those reported previously [14], further confirming that the nanoparticles had a core (cerium oxide)-shell (polymer) structure, with the polymer being PVP or a PVP-related one. Using transmission electron microscopy, it was determined that the core was almost free of PVP. This was because the core had a dense structure [12] and the thickness of the outer shell was about 10 nm [11,12].

The thick films obtained after the firing of the substrates at 900–1,000 °C had a porous structure, which consisted of spherical nanoparticles (Figure 1(b,c)). The average size of the nanoparticles at both 900 °C and 1,000 °C was approximately 120 nm, and it decreased as the substrates were heated to 900–1,000 °C. This was attributed to the decomposition of the shell structure after the heat treatment at temperatures 900 °C or higher, which suggested that after the thick films had been fired, the constituent nanoparticles did include a core.

For the XRD analyses, we prepared not only thick films fired at 900–1,000 °C but also a thick film that was fired at 800 °C. The results of the XRD analyses indicated that all the fired thick films comprised a single phase, that of cerium oxide. In addition, the crystallites of the film heated at 800 °C were greater than 30 nm in size. The full width at half maximum of the peaks of the diffraction patterns of the thick films decreased with an increase in the heat-treatment temperature. This meant that the size of the crystallites of the heated thick films also increased with an increase in the heat-treatment temperature. Figure 1(d) shows an image of a thick film prepared from the precipitated nanoparticles. The film was heated at 950 °C because heating at this temperature elicited a better response than did heating at 800 °C or at 1,100 °C [7].

**Figure 2.**
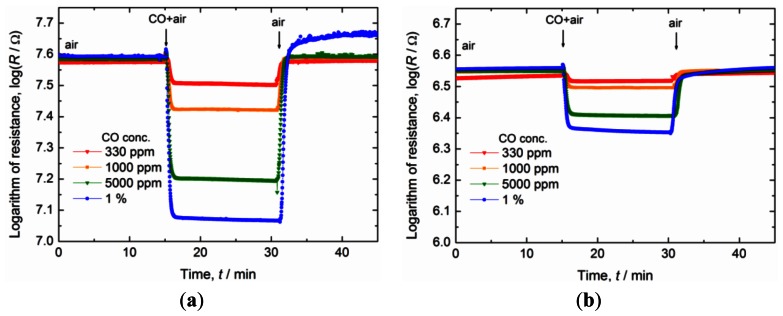
Change in the resistance of the sensors based on cerium oxide thick films (**a**) prepared from the core-shell nanoparticles and fired at 900 °C (**b**) prepared from the precipitated nanoparticles and fired at 950 °C. The sensors were exposed interchangeably to both air containing CO and CO-free air, with the operating temperature being 450 °C.

Figure 2 shows the typical responses of a sensor fabricated using the core-shell nanoparticles-based thick film that was fired at 900 °C (Figure 1(b)) and those of a sensor fabricated using the precipitated nanoparticles-based thick film that was fired at 950 °C (Figure 1(d)). The responses were measured in air containing CO in various concentrations, with the operating temperature being 450 °C. After switching to air containing CO, the resistance between the Pt electrodes in the sensors decreased. On switching back to CO-free air, the resistance returned to approximately its original value. These response curves were almost the same as those reported earlier [6,7].

A CO molecule reacts with an oxide ion in the cerium oxide lattice [6] in the following manner:

(2) $$\text{CO}={{\text{O}}_{\text{o}}}^{\text{x}}={\text{CO}}_{2}+{{\text{V}}_{\text{o}}}^{¨}+2{\text{e}}^{\prime }$$

where O_O_^X^, V_O_**^¨^**, and e′ denote an oxide ion, an oxygen vacancy, and an electron in cerium oxide, respectively. Thus, the resistance between the electrodes decreases when CO comes in contact with cerium oxide. The oxygen vacancies thus created in cerium oxide and the electrons released thereby (V_O_**^¨^** and e′, respectively) diffuse rapidly through the cerium oxide lattice. Therefore, the resistance of not only the surface but also of the entire particle decreases.

The resistance in the case of the sensor fabricated using the core-shell nanoparticles was 40 MΩ at 450 °C in air and more than 10 times higher than that of the sensor fabricated using the precipitated nanoparticles, which was 3 MΩ. The response and recovery times of the sensors, determined from Figure 2, are listed in Table 1. The response times of both the sensors were about the same though (50 s). In this study, the equipment used to switch between the air containing CO and the CO-free air introduced a time delay. The response time of sensors prepared using precipitated nanoparticles was found to be about 2 s at 450 °C when equipment capable of detecting fast response times was used [7]. Hence, these results suggested that, in this study, it took about 50 s to switch between the two types of air samples tested. In addition, the results also implied that the sensor prepared using the core-shell nanoparticles would exhibit a high response speed similar to that of the sensor fabricated using the precipitated nanoparticles, namely of 2 s, if equipment with fast switching capability were to be used. Finally, the recovery times of the two sensors, which were also approximately 50 s, were the same as their response times and much smaller than those of gas sensors based on tin dioxide [18–21].

**Figure 3.**
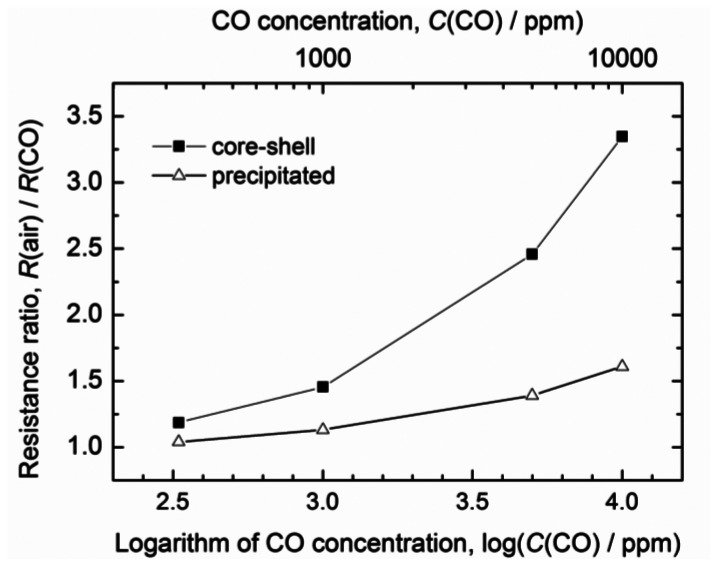
Relationship between the CO concentration in the air sample being tested and the responses of the two types of sensors at 450 °C. core-shell: sensor based on a thick film prepared from the core-shell nanoparticles and fired at 900 °C; precipitated: sensor based on a thick film prepared from the precipitated nanoparticles and fired at 950 °C.

Figure 3 shows the relationship between the response, *S*, and the CO concentration in the tested air sample at an operating temperature of 450 °C. For comparison, the response curve of the sensor fabricated using the precipitated nanoparticles is also shown. The response of the sensor based on the core-shell nanoparticles was markedly higher than that of the sensor prepared using the precipitated nanoparticles. The reason for this significant difference in the two responses is discussed later.

**Figure 4.**
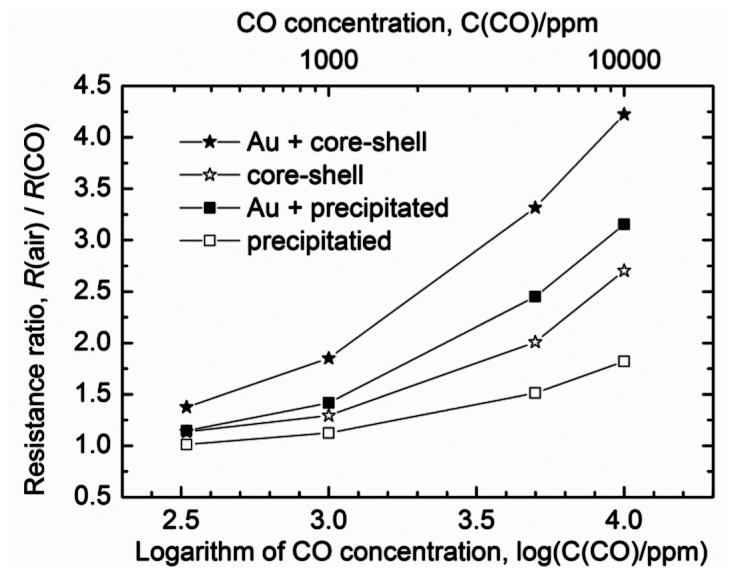
Relationship between the CO concentration in the tested air sample and the responses of various sensors at 450 °C. Au + core-shell: sensor based on a thick film formed from the core-shell nanoparticles. The thick film was fired at 1,000 °C and Au nanoparticles subsequently incorporated into it; core-shell: sensor based on a thick film prepared from the core-shell nanoparticles and fired at 1,000 °C; Au + precipitated: sensor based on a thick film formed from the precipitated nanoparticles. The thick film was fired at 950 °C and Au nanoparticles subsequently incorporated into it; and precipitated: sensor based on a thick film prepared from the precipitated nanoparticles and fired at 950 °C.

Figure 4 shows the relationship between the CO concentration and the responses of sensors based on the thick films formed using the Au colloid as well as those formed without it. These thick films were prepared from the core-shell nanoparticles and were fired at 1,000 °C. The operating temperature for these sensors was 450 °C. For comparison, the response curves of the sensors based on the precipitated nanoparticles, formed by firing at 950 °C, are also shown in Figure 4. The response of the sensors based on the thick films containing Au was higher than that of the sensors with the Au-free thick films. This effect of the addition of Au was similar to what has been reported previously [17]. That is to say, the addition of Au resulted in an increase in the responses of both types of sensors investigated. This was due to the strong reaction between the Au atoms and the oxygen vacancies [17,22]. We also checked the reproducibility of the responses of the sensors. About three to five measurements were made with a sensor prepared from the core-shell nanoparticles, with the sensors exhibiting the same respective response. This was indicative of good reproducibility.

Next, we discuss the reason why the response of the sensors fabricated using the core-shell nanoparticles was higher than that of the sensors made using the precipitated nanoparticles. First, in order to determine the effect of microsized pores on the response of the sensors, we measured the BET specific surface areas of the two nanoparticle powders. The obtained results are listed in Table 2. Powders obtained after heating the core-shell nanoparticles at 1,000 °C and the precipitated nanoparticles at 950 °C were used to determine the BET specific surface areas. These heat treatments were similar to those used to treat the thick films during the preparation of the sensors. The BET specific surface area of the powder comprising core-shell nanoparticles was approximately 7 m^2^·g^−1^ and smaller than that of the powder containing precipitated nanoparticles, which was 14 m^2^·g^−1^. That is to say, the powder of the core-shell nanoparticles contained fewer microsized pores than did the powder consisting of the precipitated nanoparticles. It was, therefore, concluded that the increase in the response of the core-shell nanoparticles-based sensor was not attributable to microsized pores. The thick film formed from the core-shell nanoparticles had larger pores than did the film formed from the precipitated nanoparticles, as shown in Figure 1, allowing the tested gas to diffuse more readily. Owing to the larger pores, CO molecules could reach the cerium oxide particles deep within the thick film formed from the core-shell nanoparticles. This meant that the entire thick film reacted with CO, resulting in the larger response.

As mentioned previously, the resistance of the sensor fabricated using the precipitated nanoparticles at 450 °C in air was 3 MΩ. This value was an order of magnitude smaller than that of the sensor based on the core-shell nanoparticles. One of the reasons for this marked difference in the resistances of the two sensors was the difference in the densities of the thick films used to form the sensors. However, a difference in resistance of an order of magnitude cannot be explained by a difference in density alone. It is assumed that the cerium oxide bulk and/or grain boundary resistances of the two sensors were also different. These differences in electrical characteristics might be related to the improvement in sensor response. In the future, we will investigate the various electrical properties, such as carrier density, mobility, bulk resistance, and grain boundary resistance, among others, of the thick films, to elucidate the causes for the improvement noticed in sensor response.

## 4. Conclusions

The response of the sensor based on the thick film prepared from the core-shell nanoparticles was higher than that of the sensor based on the thick film prepared from the precipitated nanoparticles. It was found this difference in the responses was not related to the BET specific surface areas of the thick films. An improvement in sensor response value was also noticed on the addition of Au nanoparticles to the thick films used to fabricate the two types of sensors.

## Figures and Tables

**Table 1.** Response and recovery times of the sensors.

**Type of Nanoparticles**	**90% Response Time, *t*_90,res_ (s)**	**90% Recovery Time, *t*_90,rec_ (s)**
Core-shell nanoparticles	46	55
Precipitated nanoparticles	49	52

**Table 2.** BET specific surface areas of the cerium oxide powders obtained after heating the core-shell and the precipitated nanoparticles.

**Sample Prepared From**	**Heat-Treatment Temperature (°C)**	**BET Specific Surface Area (m^2^·g^−1^)**
Core-shell nanoparticles	1,000	7 ± 2
Precipitated nanoparticles	950	14 ± 4
